# Influence of smoking cessation drugs on blood pressure and heart rate in patients with cardiovascular disease or high risk score: real life setting

**DOI:** 10.1186/s12872-015-0180-4

**Published:** 2016-01-05

**Authors:** André Pacheco Silva, Jaqueline Scholz, Tania Ogawa Abe, Gabriela Gouveia Pinheiro, Patricia Viviane Gaya, Alexandre Costa Pereira, Paulo Caleb Junior Lima Santos

**Affiliations:** Heart Institute University of Sao Paulo Medical School, Sao Paulo, Brazil; Smoking Cessation Program Department, Heart Institute (InCor), University of Sao Paulo Medical School, Av. Dr. Eneas de Carvalho Aguiar 44, Cerqueira Cesar, 05403-900 Sao Paulo, SP Brazil; Laboratory of Genetics and Molecular Cardiology, Heart Institute (InCor), University of Sao Paulo Medical School, Sao Paulo, Brazil

**Keywords:** Smoking cessation, Nicotine replacement therapy, Bupropion, Varenicline, Cardiovascular disease, Hypertension

## Abstract

**Background:**

Smoking is the most important reversible cardiovascular risk factor. It is well established that quitting smoking reduces coronary events. However, on several occasions, the cardiovascular safety of smoking cessation drugs has been questioned. Our goal is to evaluate the effects of smoking cessation drugs on blood pressure and heart rate in patients from a smoking cessation service in a cardiology hospital.

**Methods:**

We examined the PAF database (Smoking Cessation Assistance Program database) between January 2008 and March 2014. We analyzed data from 900 patients who were compliant with the treatment (50.5 % male, average age 53 ± 17 years). The most frequent clinical diagnoses were coronary artery disease (25.2 %), hypertension (57.2 %), and diabetes (13.4 %). Blood pressure, heart rate, and carbon monoxide (CO) concentration in exhaled air were analyzed at consecutive visits during the first 45 days of treatment (mean visits - 3). Analysis of repeated measures was used for the statistical analysis (*p* < 0.05).

**Results:**

Two hundred seventy one patients used nicotine replacement therapy (NRT) alone, 81 used bupropion alone, 154 used varenicline alone, 283 used NRT plus bupropion and 111 used bupropion plus varenicline. For all smoking cessation drugs, used alone or in combination, no increase occurred in the average value of systolic blood pressure (SBP), diastolic blood pressure (DBP) and heart rate (HR). Significant reductions in CO concentrations occurred in all smoking cessation drug groups.

**Conclusion:**

Smoking cessation drugs used in monotherapy or in combined regimens did not influence systolic blood pressure (SBP), diastolic blood pressure (DBP) and heart rate (HR) in this group of patients during the observation period.

## Background

Smoking cessation substantially lowers the risk of death in patients of all ages [1], including those with cardiovascular disease [2]. The long-term cardiovascular benefits of smoking cessation are well established [1, 2]. Clinical practice guidelines [3] recommend the use of cessation pharmacotherapy for smokers interested in quitting. The first-line drugs for smoking cessation are nicotine replacement therapy (NRT - gum or patch), bupropion (Zyban, Wellbutrin) and varenicline (Chantix, Champix).

These medications demonstrated effectiveness in achieving complete smoking cessation [3], but their safety in patients with cardiovascular (CV) disease has been questioned and evaluated in several studies and meta-analyses. Previous reports associated NRT use with occurrence of cardiovascular events, such as myocardial infarction, especially in patients who continued smoking [4]. However, two recent meta-analyses showed an increase in CV symptoms, including tachycardia and nonspecific chest pain [5, 6] but not in MACEs - defined as cardiovascular death, nonfatal myocardial infarction and nonfatal stroke [6]. With bupropion, trials including smokers with cardiovascular disease did not report a statistically significant increase in CV events compared to placebo. However, the sample sizes were small and not powered for safety [7–9].

A recent network meta-analysis of 21 randomized clinical trials (RCTs) showed that bupropion was not harmful for MACEs and appeared to have a cardioprotective effect [6]. Varenicline, an alpha-4 beta-2 nicotinic acetylcholine receptor partial agonist, binds to the same receptors as nicotine, which is known to have sympathomimetic cardiovascular effects [10]. The first meta-analysis on the CV safety of varenicline, predominantly composed of trials excluding patients with CV disease, concluded that increased risk (72 %) exists for minor and major cardiovascular events among tobacco users [11]. A more recent large meta-analysis with more than 9,000 patients, however, found no significant risk of major CV events associated with varenicline use [6, 12]. Trials that included patients with a history of CV disease found that even in a higher risk population, varenicline did not add significant damage [6, 12].

There are few studies evaluating combination of therapies for smoking cessation [13–15], none of them evaluating safety in patients with cardiovascular disease. One of these studies excluded patients with cardiovascular diseases or history of hypertension [15]. In the other studies there were not reports of major or minor adverse cardiovascular events during the smoking cessation therapy [13, 14]. There is not meta-analysis available in the literature until this moment.

We examined CV effects of these drugs on blood pressure and heart rate in outpatient setting according to different combination therapies and the previous presence of hypertension, coronary artery disease, and/or acute myocardial infarction.

## Methods

We analyzed 900 outpatient subjects followed at the Smoking Cessation Program of the Heart Institute (InCor), University of Sao Paulo Medical School, Sao Paulo, Brazil. Most of the patients have cardiovascular diseases or high risk score. We used the PAF database (Smokers Assistance Program) between January 2008 and March 2014 and looked up subjects of both genders older than 18 years of age. The Ethics Committee for Research Project Evaluation (CAPPesq) of the Hospital das Clinicas – School of Medicine, University of Sao Paulo, approved the study. The study complied with the Declaration of Helsinki. They agreed with the informed consent form.

During the study period, 1791 patients were enrolled to the service. However, 891 patients were excluded because they did not come in the consecutive visits and their data for BP, HR, and/or CO were not available. However, patients with analyzed data (*n* = 900) did not have significant differences in their SBP, DBP, HR and CO values (125 ± 21 mmHg; 76 ± 12 mmHg; 74 ± 13 bpm; 11 ± 10 ppm) compared with data of the untreated patients (*n* = 891) (123 ± 22 mmHg; 74 ± 18 mmHg; 74 ± 18 bpm; 12 ± 12 ppm) (*p* > 0.05).

Thus, 900 patients were included in this analysis. They received smoking cessation medication to be taken for at least 12 weeks. Among the available medications, there were nicotine replacement therapies (NRT — patch and gum), bupropion, and varenicline. In the first visit (baseline), the patient was not in use of any drug for smoking cessation. One of these drugs was prescribed as monotherapy according to the nicotine dependence level, previous use of smoking cessation drugs, availability of the medication and lack of absolute contraindication related to each drug. Varenicline was prescribed for patients who failed in previous attempts with NRT and/or bupropion, or who smoked one or more packs of cigarettes per day.

The gap between follow-up visits was 2 to 3 weeks. In all visits the patient were questioned about smoking status, compliance and collateral effects of the medication prescribed, and we evaluated the vital signs, CO level, and withdrawal symptoms.

In the second visit, the patients who achieved success with monotherapy and had no or mild withdrawal symptoms were kept with the same medication. The patients who did not achieve success and the patients who achieved complete cessation but with moderate or intense withdrawal symptoms had associated therapies [13].

The data were categorized in 3 visits in sequential follow-up, called initial (baseline), second, and third visits. Variables from the database included in the analysis were: age; sex; smoking cessation; smoking cessation drugs used; smoker’s degree of nicotine dependence according to the Fagerström score [16] and Issa score [17] at the initial visit; presence or absence of hypertension, diabetes mellitus type 2 (DMT2) and coronary artery disease (CAD); acute myocardial infarction (AMI); obesity, heart failure, arrhythmias, or valvular heart disease; and the number of cardiovascular medications being used at the initial visit. All the visits and measurements were done during the afternoon period. In each visit, the median of 2 blood pressures was taken with a mercury sphygmomanometer, and heart rate and exhaled CO level were collected. The parameter we used for smoking cessation was CO level below 6 ppm [18] associated with the self-reported cessation. The data regarding systolic blood pressure (SBP), diastolic blood pressure (DBP), and heart rate (HR) of study patients were compared for initial, second, and third visits, according to smoking cessation drugs used, alone or in combination, as well as between 2 groups: first with the presence of hypertension, CAD, and/or AMI and second without all these diseases.

### Statistical analysis

Categorical variables are presented as percentages and continuous variables are presented as means ± SD (standard deviation). Friedman’s test (repeated measures) was performed to analyze SBP, DBP, HR, and CO levels during visits, according to the prescribed pharmacotherapy and the presence of hypertension, CAD, or AMI. Friedman’s test was also performed to analyze SBP, DBP, and HR according with the CO cutoff (6 ppm) in the second and third visits and the presence of hypertension, CAD, or AMI. Statistical analyses were carried out using SPSS 16.0 software (IBM, New York, NY), with the level of significance set at *p* < 0.05.

## Results

During the study, 900 patients were included in the analysis. The median age of patients was 53 ± 17 and 50.5 % were male. Table 1 shows demographic and clinical characteristics. Smoker’s degree of nicotine dependence was 6.3 ± 2.3 assessed by Fagerström score [16] and 2.9 ± 0.9 by Issa score [17]. From the 900 patients included, 580 (64.4 %) had hypertension, CAD, and/or AMI. Hypertension was the most common cardiovascular disease (57.2 %), and a significant number of patients had coronary artery disease (25.2 %) and previous acute myocardial infarction (27.0 %).

**Table 1:** Demographic and clinical characteristics of study patients

Variables	*n* = 900
Sex, female (%)	49.5
Fagerström score	6.3 ± 2.3
Issa score	2.9 ± 0.9
Hypertension (%)	57.2
Coronary artery disease (%)	25.2
Acute myocardial infarction (%)	27.0
Diabetes mellitus type 2 (%)	13.4
Obesity (%)	9.1
Number of medicines	3.6 ± 3.4

Among these 900 patients, 271 patients used NRT alone, 81 used bupropion alone, 154 used varenicline alone, 283 used NRT + bupropion, and 111 used bupropion + varenicline.

Table 2 shows the sequential measures of blood pressures, heart rate and CO concentration of patients according to prescribed drugs and the presence of hypertension, CAD, or AMI. We observed that SBP, DBP, and HR did not change significantly (*p* > 0.05) during 3 sequential measures in follow-up, regardless of pharmacotherapy for smoking cessation used and the presence of hypertension, CAD, and/or previous AMI. The CO analysis showed a significant reduction (*p* < 0.001) in exhaled CO level in 3 sequential measures in follow-up in all the types of pharmacotherapy for smoking cessation used, alone or in combination.

**Table 2:** Sequential measures of blood pressures, heart rate, and monoximetria of patients according to prescribed drugs and the presence of hypertension, CAD, or AMI

Varenicline		
	Presence of hypertension, CAD, or AMI	
Variables	No (*n* = 85)	Yes (*n* = 69)
Initial SBP (mmHg)	118 ± 14	131 ± 21
Second visit SBP (mmHg)	117 ± 13	127 ± 20
Third visit SBP (mmHg)	117 ± 15	128 ± 19
*p* value	0.20	0.10
Initial DBP (mmHg)	76 ± 10	81 ± 12
Second visit DBP (mmHg)	74 ± 8	78 ± 10
Third visit DBP (mmHg)	76 ± 10	79 ± 11
*p* value	0.26	0.16
Initial HR (bpm)	76 ± 11	76 ± 10
Second visit HR (bpm)	75 ± 9	76 ± 10
Third visit HR (bpm)	75 ± 8	74 ± 10
*p* value	0.60	0.30
Initial monoximetria (ppm)	15 ± 11	12 ± 7
Second visit monoximetria (ppm)	8 ± 10	7 ± 6
Third visit monoximetria (ppm)	4 ± 9	4 ± 5
*p* value	<0.001	<0.001
Varenicline Plus Bupropione		
	Presence of hypertension, CAD, or AMI	
Variables	No (*n* = 62)	Yes (*n* = 49)
Initial SBP (mmHg)	117 ± 14	128 ± 18
Second visit SBP (mmHg)	117 ± 15	126 ± 21
Third visit SBP (mmHg)	115 ± 15	129 ± 21
*p* value	0.56	0.63
Initial DBP (mmHg)	75 ± 8	81 ± 12
Second visit DBP (mmHg)	74 ± 8	81 ± 12
Third visit DBP (mmHg)	74 ± 8	81 ± 11
*p* value	0.85	0.27
Initial HR (bpm)	79 ± 10	73 ± 10
Second visit HR (bpm)	74 ± 13	73 ± 10
Third visit HR (bpm)	77 ± 10	73 ± 11
*p* value	0.06	0.86
Initial monoximetria (ppm)	17 ± 10	16 ± 8
Second visit monoximetria (ppm)	11 ± 10	10 ± 7
Third visit monoximetria (ppm)	7 ± 7	9 ± 8
*p* value	<0.001	<0.001
Bupropione		
	Presence of hypertension, CAD, or AMI	
Variables	No (*n* = 39)	Yes (*n* = 42)
Initial SBP (mmHg)	112 ± 14	132 ± 27
Second visit SBP (mmHg)	117 ± 17	126 ± 23
Third visit SBP (mmHg)	116 ± 15	132 ± 28
*p* value	0.12	0.31
Initial DBP (mmHg)	74 ± 7	81 ± 12
Second visit DBP (mmHg)	76 ± 10	78 ± 10
Third visit DBP (mmHg)	76 ± 9	82 ± 11
*p* value	0.63	0.14
Initial HR (bpm)	76 ± 10	73 ± 13
Second visit HR (bpm)	76 ± 8	73 ± 14
Third visit HR (bpm)	75 ± 9	74 ± 14
*p* value	0.25	0.99
Initial monoximetria (ppm)	14 ± 13	12 ± 11
Second visit monoximetria (ppm)	7 ± 10	8 ± 6
Third visit monoximetria (ppm)	6 ± 8	7 ± 6
*p* value	<0.001	<0.001
Bupropione Plus NRT		
	Presence of hypertension, CAD, or AMI	
Variables	No (*n* = 72)	Yes (*n* = 211)
Initial SBP (mmHg)	117 ± 16	132 ± 21
Second visit SBP (mmHg)	117 ± 18	134 ± 22
Third visit SBP (mmHg)	118 ± 18	132 ± 21
p value	0.89	0.53
Initial DBP (mmHg)	73 ± 10	80 ± 12
Second visit DBP (mmHg)	73 ± 11	79 ± 13
Third visit DBP (mmHg)	74 ± 11	80 ± 13
*p* value	0.67	0.81
Initial HR (bpm)	76 ± 11	73 ± 14
Second visit HR (bpm)	75 ± 13	74 ± 12
Third visit HR (bpm)	75 ± 11	74 ± 13
*p* value	0.87	0.83
Initial monoximetria (ppm)	11 ± 7	11 ± 8
Second visit monoximetria (ppm)	7 ± 6	7 ± 8
Third visit monoximetria (ppm)	5 ± 6	5 ± 5
*p* value	<0.001	<0.001
NRT		
	Presence of hypertension, CAD, or AMI	
Variables	No (*n* = 62)	Yes (*n* = 209)
Initial SBP (mmHg)	115 ± 17	130 ± 23
Second visit SBP (mmHg)	119 ± 18	128 ± 23
Third visit SBP (mmHg)	116 ± 17	130 ± 22
p value	0.48	0.65
Initial DBP (mmHg)	72 ± 11	78 ± 14
Second visit DBP (mmHg)	74 ± 13	77 ± 13
Third visit DBP (mmHg)	73 ± 13	77 ± 13
*p* value	0.78	0.24
Initial HR (bpm)	75 ± 11	72 ± 13
Second visit HR (bpm)	74 ± 10	72 ± 14
Third visit HR (bpm)	72 ± 10	72 ± 14
*p* value	0.10	0.90
Initial monoximetria (ppm)	10 ± 7	10 ± 6
Second visit monoximetria (ppm)	6 ± 5	5 ± 5
Third visit monoximetria (ppm)	4 ± 4	5 ± 5
*p* value	<0.001	<0.001

*NRT* nicotine replacement therapy (patch and/or gum), *CAD* coronary artery disease, *AMI* acute myocardial infarction, *SBP* systolic blood pressure, *DBP* diastolic blood pressure, *HR* heart rate

We also analyzed BP and HR of followed patients (*n* = 900) according with a CO cutoff of 6 ppm in the second and third visits and the presence of hypertension, CAD, or AMI. There was no significant influence of this variable in change BP and HR in all smoking cessation drugs group.

## Discussion

It is well known that quitting smoking can reduce the risk of mortality from heart disease and acute myocardial infarction [2]. However, studies regarding CV risks associated with pharmacotherapies for smoking cessation have been concerned with public health. Few randomized controlled trials have been conducted in populations with high cardiovascular risk profiles and also few trials have compared head-to-head the safety of smoking cessation medications.

We did not observe any significant change in systolic or diastolic blood pressure and heart rate in 900 patients who used smoking cessation therapies and were followed at Heart Institute, a cardiac hospital with a large rate of patients with hypertension, CAD, and/or AMI. This result was regardless of the combination of pharmacotherapies used in the smoking cessation treatment and the presence or absence of cited comorbidities.

In the literature, tachycardia is a well-established and benign adverse effect observed in a lot of studies using NRT [5]. Some studies showed an increase in HR (10–15 beats/min) and blood pressure (5–10 mmHg) after NRT use [19, 20], even in smokers who interrupted tobacco consumption [20]. A recent meta-analysis showed similar results - an association of using NRT and increasing minor CV events, mainly due to the occurrence of tachycardia, but without adding risk to major CV events [6]. In patients with a history of a predisposing high-risk condition, in a smaller sample, this was not found [6]. The development of tolerance to blood pressure effects of nicotine in heavy smokers can be included in these divergent hemodynamic findings.

Concerning bupropion, an increase in blood pressure could be an important cardiovascular side effect, regardless of pre-existing hypertension, because of the effect of bupropion on the reuptake of norepinephrine [21]. However, this effect is not seen overall in clinical practice and our results are in accordance with the results from other studies. Settle et al, enrolling more than 500 patients with depression, did not show clinically important effects on BP or HR when bupropion was compared with placebo [22]. In patients with CV diseases, a randomized trial including 248 smokers hospitalized because of CAD did not find a significant change in blood pressure after 12 weeks of slow release (SR) bupropion compared with placebo [7]. A clinical trial with 629 persistent smokers with CV disease, in which nearly half had experienced an AMI previously, evaluated bupropion versus placebo and did not find significant changes in BP or HR. Patients with baseline BP ≥ 160/100 mmHg were excluded [8]. In another study with 300 outpatient smokers with untreated stage 1 hypertension, different doses of bupropion induced a small reduction in BP, and bupropion 400 mg SR also increased heart rate by 2.9 beats/min versus placebo [23]. On the other hand, studies by Roose et al with small samples observed that bupropion can increase sympathomimetic activity and increase HR and BP in patients with depression and heart disease if it is used in higher doses [24, 25].

In the same way, Tonstad et al reported no changes in BP after 24 weeks of varenicline versus placebo. The mean HR remained similar in the varenicline group and decreased by 2 beats per minute in the placebo group [26]. Additionally, a randomized trial with 714 smokers with stable CV disease (history of myocardial infarction, coronary revascularization, angina pectoris, peripheral arterial vascular disease, stroke or transient ischemic attack) showed a 0.5 mmHg increase in SBP and no changes in DBP and HR in the varenicline group versus placebo [10].

There are some studies with the use of NRT in population of smokers without cardiovascular diseases or with hypertension alone [27, 28], which did not find changes in HR and BP.

This study has some limitations. BP and HR were measured at 3 consecutive visits. Therefore, although it is enough to have a comparison, possible small changes might be seen in a more prolonged follow. Compliance with the treatment and smoking status were measured during the follow-up period by asking patients and by measuring exhaled CO level, which showed a significant reduction in comparison with basal CO level, regardless of pharmacotherapy used. It indicates reduction or cessation of smoking. We did not design a study with the objective and power to observe major CV events.

Our findings of maintenance of BP and HR in a relatively large population of smokers with and without preexisting stable CV diseases using different combinations of smoking cessation therapies are important as a safety data for clinical practice. This result is in agreement with a recent meta-analysis of the use of NRT, bupropion and varenicline, which did not find an increase in major CV events with all drugs [6]. In patients with CV diseases, the existing data do not suggest harm and the benefits of smoking cessation in the long term exceeds an eventual small risk associated with pharmacotherapy drugs for smoking cessation.

## Conclusion

Our data from a real clinical practice suggest that, even in patients with hypertension, CAD, and/or AMI, there are no significant clinical change in blood pressure or heart rate during the use of NRT, varenicline, and/or bupropion.

## Abbreviations

AMI: acute myocardial infarction
BP: blood pressure
CAD: coronary artery disease
CV: cardiovascular
DBP: diastolic blood pressure
HR: heart rate
MACE: Major Adverse Cardiovascular Event
NRT: nicotine replacement therapy
PAF: Program of Assistance to Smokers
SBP: systolic blood pressure
